# Microelectrical Impedance Spectroscopy for the Differentiation between Normal and Cancerous Human Urothelial Cell Lines: Real-Time Electrical Impedance Measurement at an Optimal Frequency

**DOI:** 10.1155/2016/8748023

**Published:** 2016-02-21

**Authors:** Yangkyu Park, Hyeon Woo Kim, Joho Yun, Seungwan Seo, Chang-Ju Park, Jeong Zoo Lee, Jong-Hyun Lee

**Affiliations:** ^1^Department of Medical System Engineering, Gwangju Institute of Science and Technology (GIST), MEMS and Nano Systems Lab. #317, Dansan Building, 123 Cheomdan-gwagiro, Buk-gu, Gwangju 500-712, Republic of Korea; ^2^School of Mechatronics, Gwangju Institute of Science and Technology (GIST), MEMS and Nano Systems Lab. #317, Dansan Building, 123 Cheomdan-gwagiro, Buk-gu, Gwangju 500-712, Republic of Korea; ^3^Department of Urology, Pusan National University Hospital, 179 Gudeok-ro, Seo-gu, Busan 602-739, Republic of Korea

## Abstract

*Purpose*. To distinguish between normal (SV-HUC-1) and cancerous (TCCSUP) human urothelial cell lines using microelectrical impedance spectroscopy (*μEIS*).* Materials and Methods*. Two types of *μEIS* devices were designed and used in combination to measure the impedance of SV-HUC-1 and TCCSUP cells flowing through the channels of the devices. The first device (*μEIS-OF*) was designed to determine the optimal frequency at which the impedance of two cell lines is most distinguishable. The *μEIS-OF* trapped the flowing cells and measured their impedance at a frequency ranging from 5 kHz to 1 MHz. The second device (*μEIS-RT*) was designed for real-time impedance measurement of the cells at the optimal frequency. The impedance was measured instantaneously as the cells passed the sensing electrodes of *μEIS-RT*.* Results*. The optimal frequency, which maximized the average difference of the amplitude and phase angle between the two cell lines (*p* < 0.001), was determined to be 119 kHz. The real-time impedance of the cell lines was measured at 119 kHz; the two cell lines differed significantly in terms of amplitude and phase angle (*p* < 0.001).* Conclusion*. The *μEIS-RT* can discriminate SV-HUC-1 and TCCSUP cells by measuring the impedance at the optimal frequency determined by the *μEIS-OF*.

## 1. Introduction

Cystoscopy is considered the gold standard test for urothelial cancer of the bladder (UCB). Unfortunately, the invasiveness of this test leads to discomfort and pain during the procedure, and as a result, many patients are hesitant and fearful of the examination [1]. The procedure may also cause transient complications, such as hematuria and urethral injury, as well as dysuria. Voided urine cytology (VUC), on the other hand, is the most established noninvasive technique with high specificity (up to 99%) for the detection of UCB. However, the application of VUC is limited to an adjunct test for cystoscopy in clinical practice due to its low sensitivity (below 40%) [2]. Recently, the development of an accurate noninvasive test that may reduce the need for cystoscopy has become an area of interest. These noninvasive tests may reduce patient discomfort and their reluctance to undergo checkups for the disease, which thereby improves the accessibility of UCB examination.

Electrical impedance spectroscopy (*EIS*) is a sensitive analytical tool that characterizes the electrical properties of different types of cells [3, 4]. Electrical impedance, which refers to a typical electrical property, consists of the amplitude and phase angle. The amplitude of the impedance is the absolute value of the voltage-to-current ratio, whereas the phase angle is the phase delay between the current and voltage signals [5]. Recently, several studies introduced microelectrical impedance spectroscopy (*μEIS*) devices to differentiate between normal and cancer cells using their amplitudes and phase angles [6–9]. This has been demonstrated to be an effective strategy for a diagnostic tool with cost effective, prompt, and noninvasive techniques without the need to tag or modify cells [6–8, 10–12].

The *μEIS* techniques can be divided into two major approaches: single-cell trapping [6, 13, 14] and flow cytometry [4, 10, 15]. The single-cell trapping method is suitable for finding the optimal frequency at which the targeted cell lines, such as normal and cancerous cells, have the highest degree of differentiation. However, its limited throughput may not be appropriate for instantaneously obtaining impedance values from a large number of cells [16]. Moreover, this method cannot capture every single cell in the specimen, and the microscope-based inspection used to confirm whether single cells are trapped is quite burdensome and time-consuming, which may limit its use in clinical applications. In contrast, flow cytometry measures the electrical impedance of every single cell in real time as they flow continuously through the microelectrodes [16]. However, the impedance of the cells can only be measured at a fixed frequency in this real-time technique [15]. Thus, it is unsuitable for determining the optimal frequency. The present study focuses on combining the two approaches of electrical impedance measurement, compensating each other to effectively discriminate different types of cells.

In this study, two types of *μEIS* devices are developed, each with a different purpose, to measure the electrical impedance of normal (SV-HUC-1) and cancerous (TCCSUP) urothelial cell lines at the single-cell level. Our goal is to investigate the effectiveness of impedance measurement using a combination of these two devices in discriminating between the two cell lines. The present study is an initial step toward our eventual plan to use *μEIS* devices in urine samples as a noninvasive supplementary tool to detect UCB and, if possible, reduce the number of invasive cystoscopic examinations.

## 2. Materials and Methods

### 2.1. Device Design

**Figure 1:**
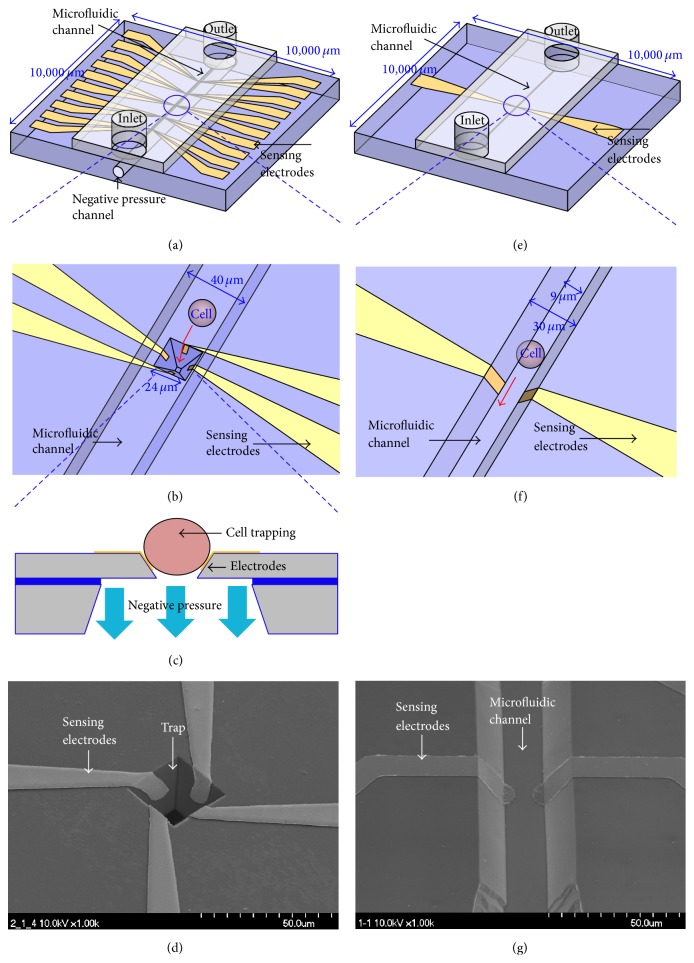
Illustration of the *μEIS *devices. (a) *μEIS-OF*: schematic view; (b) *μEIS-OF*: magnified schematic view of the sensing mechanism; (c) *μEIS-OF*: cross-sectional view of the trap mechanism; (d) *μEIS-OF*: SEM image; (e) *μEIS-RT*: schematic view; (f) *μEIS-RT*: magnified schematic view of the sensing mechanism; and (g) *μEIS-RT*: SEM image.

The first device, named *μEIS-OF*, was designed to determine the optimal frequency at which the difference in the impedance between SV-HUC-1 and TCCSUP was maximized. To this end, the electrical impedance was measured within a certain frequency range of 5 kHz–1 MHz (frequency sweeping). This device requires cells to be captured to ensure that the position of the target cells remains constant during the measurement, because frequency sweeping takes several seconds. To effectively capture the target cells, five three-dimensional traps capable of holding five cells were employed in the *μEIS-OF*. As depicted in Figure 1(a), the *μEIS-OF* consists of three parts: (a) a microfluidic channel for cell flow; (b) sensing electrodes on the sidewall of the traps; and (c) a negative pressure channel to capture the cells. Figures 1(b) and 1(c) describe the working principle of the device. While negative pressure leads the target cells to be captured in the trap, the sensing electrodes can detect the impedance of the captured cells. A SEM image of the sensing electrodes on the slanted side walls is shown in Figure 1(d).

The design of second device (named *μEIS-RT*) aimed to measure the electrical impedance of target cells at a predetermined optimal frequency in real time. Because the impedance at a specific frequency can be measured within a few milliseconds, this device does not require the cells to be captured. Thus, the impedance of flowing target cells can be measured instantaneously in real time after the optimal frequency has been determined by the *μEIS-OF*. As illustrated in Figure 1(e), the *μEIS-RT* consists of two parts: (a) a microfluidic channel and (b) sensing electrodes. In this device, cells continuously flow through the cell-flowing channel, while the impedance of the cell is measured simultaneously in a flow state using an automated real-time measurement system (Figure 1(f)). This method consequently leads to significantly faster progression of the examination compared to the *μEIS-OF*. Figure 1(g) shows a SEM image of the *μEIS-RT*.

### 2.2. Device Fabrication

**Figure 2:**
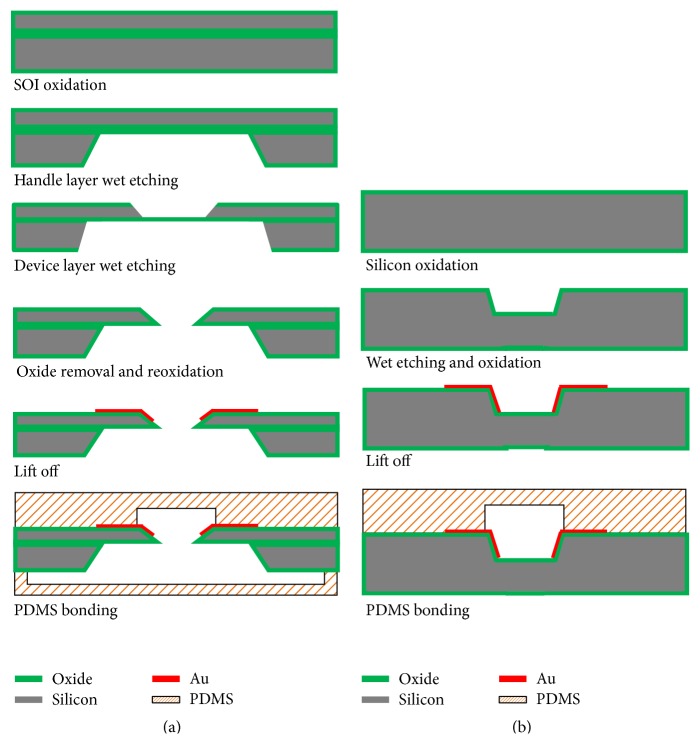
Fabrication process of the *μEIS* devices. (a) *μEIS-OF* and (b) *μEIS-RT*.

Figure 2 illustrates the fabrication process of the proposed devices. Each device was fabricated using micromachining technology and is made up of two bonded parts, silicon and PDMS layers. The detailed fabrication sequences of the two devices are as follows.

For the *μEIS-OF* (Figure 2(a)), the silicon-on-insulator (SOI) wafer was initially oxidized in a furnace. To realize a trench for a cell trap, the patterned oxide by reactive ion etching (RIE) on the handle and device layers was etched (wet etching) until the buried oxide layer was exposed. Subsequently, the buried oxide layer was eliminated by a buffered oxide etchant. After the wafer was reoxidized for the electrical passivation layer, Au/Cr for the sensing electrodes were deposited (sputtering) and these metal films were successfully patterned even on the slanted side walls by a lift-off process using a negative photoresist. To produce the cell-flowing and cell-trapping channels, PDMS stamping process was used; SU-8 molds were prepared and the PDMS replicas were detached from the SU-8 molds after curing in a convection oven. Finally, the fabricated channels layers were bonded onto the front and back of the silicon substrate. To seal the device, an oxygen plasma treatment was performed prior to the bonding between the channel layers and silicon substrate, and a silicone tube was fixed using a biocompatible bond (Henkel Loctite Corp., Rocky Hill, CT, USA).

The *μEIS-RT* (Figure 2(b)) was fabricated using a similar sequence to that of the *μEIS-OF*. The fabrication difference between two devices is that the cell-flowing channel of the *μEIS-RT* was formed using wet etching instead of PDMS stamping, and the backside wet etching is not necessary for the fabrication of the trap. Thus, the *μEIS-RT* can be simply fabricated with only two photomasks, making it suitable for mass production.

### 2.3. Experimental Setup and Process

**Figure 3:**
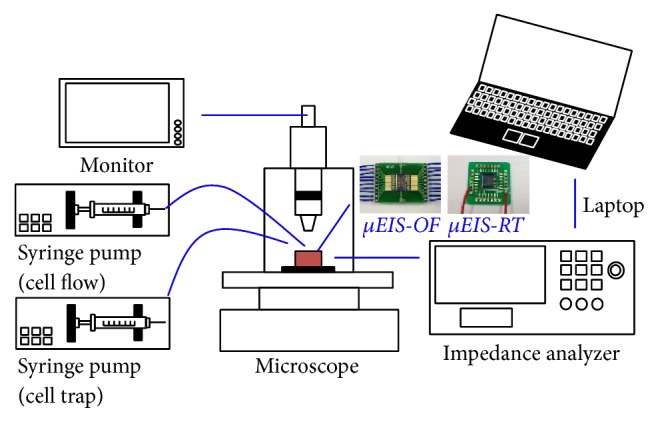
Schematic of the experimental setup for the impedance measurements in which the two devices were used. (Note that a syringe pump for the cell trap is not required during the *μEIS-RT* experiment.)

SV-HUC-1 and TCCSUP were prepared and cultured according to the general guidelines of the American Type Culture Collection, and the experimental setup for the *μEIS* devices is depicted in Figure 3. The optimal frequency was determined by coupling the *μEIS-OF* to an impedance analyzer (HP 4294A; Agilent Technologies, Santa Clara, CA, USA). Each cell line in phosphate buffered saline (PBS) was injected into the microfluidic channel of the *μEIS-OF* using a syringe pump (PHD 2000 and Pump 11 Elite; Harvard Apparatus, Holliston, MA, USA). Another identical syringe pump applied negative pressure at the *μEIS-OF* for cell trapping. The flow rates of the syringe pumps to allow cells to flow and trap cells were 1 and 0.5 *μ*L/min, respectively, and the passage of cells through the cell-flowing channel was confirmed using a microscope (Eclipse L200; Nikon Corporation, Tokyo, Japan). The impedance analyzer applied a voltage of 0.5 V_p-p_ to the *μEIS-OF* at the sweep frequency range (5 kHz–1 MHz) to determine the optimal frequency.

The *μEIS-RT* was used to measure the electrical impedance of the two cell lines in real time in a flow state. LabVIEW (National Instrument, Austin, TX, USA) was utilized for an automated real-time measurement to control the impedance analyzer and acquire raw data as text files via general-purpose interface bus (GPIB) communication. The applied voltage (0.5 V_p-p_) and sampling interval (12.5 ms) of the impedance analyzer were maintained throughout the experiments at the optimal frequency determined by the *μEIS-OF*, along with the flow rate of the syringe pump for cell flow (0.1 *μ*L/min). During cell flow, the real-time impedance of the cells was measured as they passed through the sensing electrodes.

Deionized water (DI) was used to clean the fluidic channels of the devices and each experiment was conducted within an hour to reduce the cell damage. A room temperature of 25°C was maintained throughout the experiments.

A statistical analysis was performed to compare the electrical impedance between SV-HUC-1 and TCCSUP cells: Student's *t*-test and Mann-Whitney *U* test were used to compare the impedance between the two cell lines measured by the *μEIS-OF* and the *μEIS-RT*, respectively. The results are considered significant at *p* value < 0.05. All statistical calculations were computed using PASW Statistics 18 (SPSS Inc., Chicago, IL, USA).

## 3. Results

### 3.1. Impedance Measurement to Determine the Optimal Frequency Using the *μEIS-OF*

**Figure 4:**
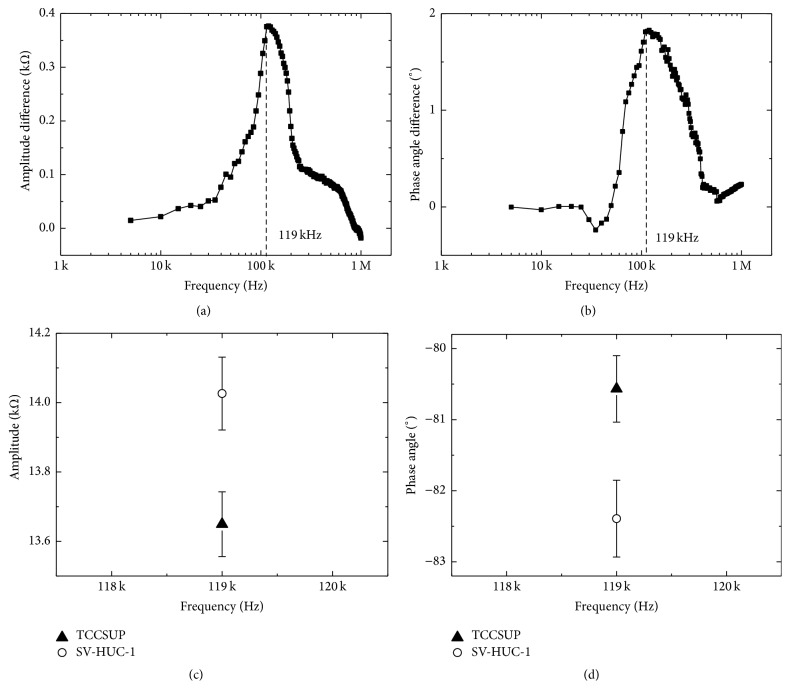
Experimental results of *μEIS-OF*. (a) Average amplitude difference and (b) average phase angle difference between the two cell lines. Optimal frequency was determined to be 119 kHz. Each value is calculated from the difference between absolute impedance values of normal and cancer cells (Amplitude  difference = |Amplitude_normal_| − |Amplitude_cancer_|, Phase  angle  difference = |Phase  angle_normal_| − |Phase  angle_cancer_|). (c) Amplitude and (d) phase angle of each cell line at 119 kHz: the vertical bar represents a standard deviation.

Thirty and fifty cells from suspensions of SV-HUC-1 and TCCSUP, respectively, were captured in the trap of the *μEIS-OF*, and the electrical impedance of each cell was measured by sweeping the frequency from 5 kHz to 1 MHz. The electrical impedance differences are dependent on the frequency of each type of urothelial cell line and are presented in Figures 4(a) and 4(b), where the values in the graphs are the difference between the absolute impedance values of normal and cancer cell lines. When comparing SV-HUC-1 with TCCSUP cells, the impedances can be distinguished in terms of the amplitude and phase angle. Specifically, maximum differences in the average impedance values between the two cell lines are observed at 119 kHz, and these differences are statistically significant (*p* < 0.001, Table 1). The mean impedance values are listed in Table 1. The difference in the amplitude and phase angle between the two cell lines at 119 kHz is 376.6 Ω and 1.8°, respectively. Figures 4(c) and 4(d) show the mean amplitude and phase angle values, respectively, of the target cell lines that are clearly discriminated at 119 kHz, including the error bars (standard deviation).

### 3.2. Real-Time Impedance Measurement at Optimal Frequency Using the *μEIS-RT*

**Figure 5:**
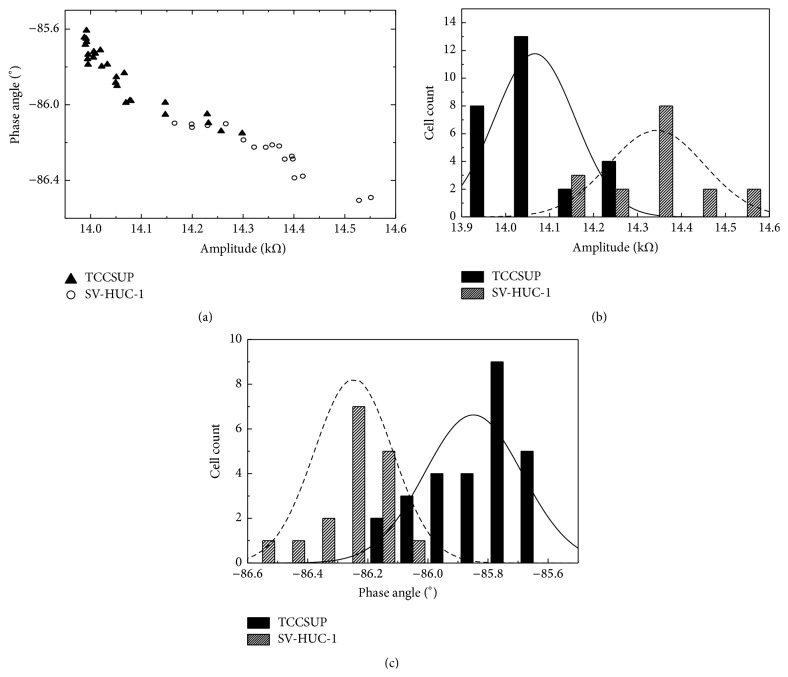
Real-time impedance measurement at 119 kHz. (a) Scatter plot of the amplitude versus the phase angle. (b) Histogram of the amplitude for the two cell lines. (c) Histogram of the phase angle for the two cell lines. Each impedance parameter in the graphs shows statistically significant differences between the two cell lines (*p* < 0.001).

The electrical impedance of seventeen and twenty-seven SV-HUC-1 and TCCSUP cells, respectively, was measured at 119 kHz by the *μEIS-RT*.  Figure 5(a) shows the scatter plot of the amplitude versus the phase angle of the two cell lines detected at 119 kHz. The absolute amplitude and phase angle values of TCCSUP tended to be lower than those of SV-HUC-1, which agrees with the *μEIS-OF* result at 119 kHz. Figures 5(b) and 5(c) show that the histograms of each cell line can be fitted with two normal distributions, which correspond to SV-HUC-1 and TCCSUP. When comparing the two cell lines, both the amplitude and phase angle can be easily distinguished, as shown in the graphs in Figure 5. Furthermore, the amplitude and phase angle of the two cell lines significantly differ at 119 kHz (*p* < 0.001, Table 1). The mean values of the impedance of each cell line at 119 kHz are summarized in Table 1.

## 4. Discussion

**Figure 6:**
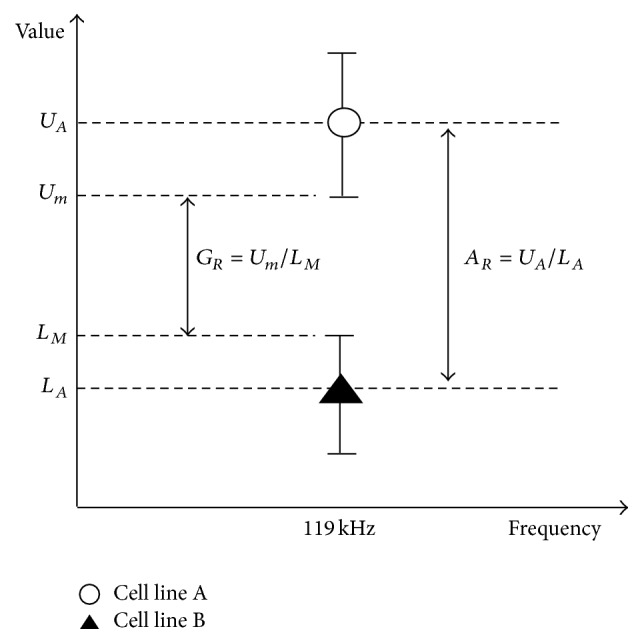
A plot showing the concept of differentiation index. The differentiation index (*D*), which is the product of the average ratio (*A*_*R*_) to the gap ratio (*G*_*R*_), was applied to evaluate the effectiveness of the *μEIS-OF* to discriminate between the two cell lines. The calculated *D* values in both amplitude and phase angle exceeded 1.0. Therefore, the cell lines were well discriminated with a low variance at 119 kHz. (*U*_*A*_: average value of the upper level, *U*_*m*_: minimum value of the upper level, *L*_*A*_: average value of the lower level, and *L*_*M*_: maximum value of the lower level).

The optimal frequency measured by the *μEIS-OF* is observed at 119 kHz in the present study. To quantitatively evaluate the ability of the *μEIS-OF* to distinguish the two target cell lines at 119 kHz, the differentiation index (*D*), as described by Kang et al. [8], was employed as follows:

(1) $$\begin{array}{c} D=G_{R}\times A_{R}=\frac{U_{m}}{L_{M}}\times \frac{U_{A}}{L_{A}}, \end{array}$$

where *D*, *G*_*R*_, *A*_*R*_, *U*_*A*_, *U*_*m*_, *L*_*A*_, and *L*_*M*_ are the differentiation index, gap ratio, average ratio, average value of the upper level, minimum value of the upper level, average value of the lower level, and maximum value of the lower level, respectively (Figure 6). The calculated *D* value at 119 kHz is 1.041 for the amplitude and 1.033 for the phase angle in the *μEIS-OF*. These values are greater than 1.0, indicating that the target cell lines, SV-HUC-1 and TCCSUP, are well distinguished with a low variance at 119 kHz using the *μEIS-OF*. This result supports the capability of the *μEIS-RT* in differentiating between SV-HUC-1 and TCCSUP cells at 119 kHz.

The electrical impedance of the two cell lines measured in real-time using the *μEIS-RT* significantly differs at a frequency of 119 kHz. Although the amplitude and phase angle of some cells overlapped, an amplitude less than 14.1 kΩ and phase angle greater than −86.0° are exclusive to the TCCSUP cells (displayed at left upper quadrant of Figure 5(a)). In other words, the cells detected in this impedance domain at 119 kHz can be considered TCCSUP cells. Moreover, the impedance of this domain may be a specific electrical property of TCCSUP that distinguishes this cell line from others. Additional comparative studies using other cell lines are necessary to verify this prediction.

The results of the present study show that 29.4% (*n* = 5) and 22.2% (*n* = 6) of SV-HUC-1 and TCCSUP cells, respectively, detected in the *μEIS-RT* overlap in terms of amplitude at 119 kHz (Figure 5(b)). In addition, the phase angle of 35.3% (*n* = 6) and 18.5% (*n* = 5) of the same respective cells overlap at 119 kHz (Figure 5(c)). This may be due to the effect of cell size variation. Electrical impedance is a complex function of cell status, which includes the cell composition, cellular membrane properties, medium surrounding the cell, and the cell size [10]. In other words, the electrical impedance is related to the size of the measured cells, as well as their physiological properties. Consequently, variations in the cell size may hinder the ability of the electrical impedance to distinguish between normal and cancerous cells [17]. Notably, the impedance of cell types with identical material properties may not be equivalent if the sizes are different. Moreover, the impedances of cells whose material properties differ is disparate for cells of the same size. This implies that the impedances may not be distinguishable if the cell sizes are different. As such, the several overlapped points between TCCSUP and SV-HUC-1 in Figure 5 could be as a result of the variation in cell size. A compensation algorithm for the cell size deviation needs to be developed in future studies.

A few studies have reported a differentiation between normal and cancerous cells. In 2007 [6], a *μEIS* device in which the cells were supposed to occupy the volume between the electrodes was introduced to differentiate between the normal and cancerous human breast cell lines. Both the amplitude and phase were clearly discriminated between the cell lines at a frequency of 100 kHz. Another study reported using a *μEIS* constriction channel to form an effective seal between the cell and the channel [18]. In this study, osteoblasts and osteocytes were classified in terms of the amplitude with a success rate of 85.3% at 100 kHz. In 2012 [7], a *μEIS* device in which the height of microfluidic channel was significantly smaller than the cell diameter was developed. The smaller channel can squeeze the flowing cells and tighten the electrical contact between the cells and sensing electrodes. The device discriminates between the normal and cancerous human breast cancer cell lines in terms of resistance and phase without overlapping at 500 kHz.

Although the present study has several advantages over the abovementioned studies, such as determining the optimal frequency to measure the real-time cell impedance, or showing the potential of actual clinical use in finding UCB using urine specimen, our devices present relatively higher overlapping data between the two cell lines. The abovementioned *μEIS* devices were commonly designed to minimize the current leakage into the extracellular media to minimize the overlapping data during cell discrimination. Therefore, maximizing the current flow into a cell, rather than the extracellular media, may be a solution to enhance the differentiation ability between different cell lines for our device.

**Figure 7:**
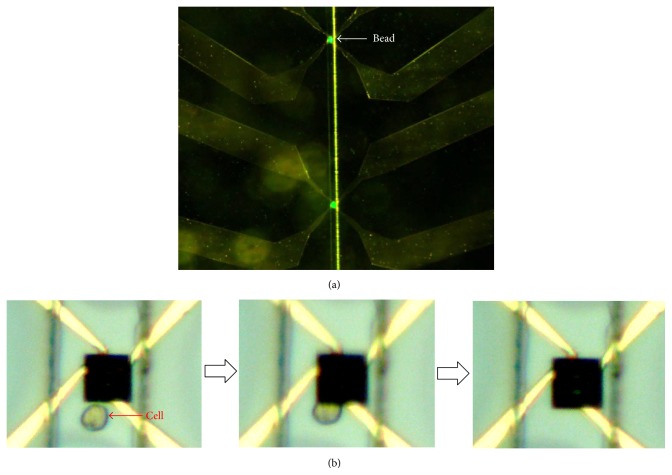
Optical microscopic images to confirm the trap of beads and cells in *μEIS-OF*. (a) Trapped fluorescent beads and (b) sequential flow of a cell via microfluidic channel into a trap.

Prior to the electrical impedance measurement using urothelial cells, the trapping efficiency of the *μEIS-OF* was tested using a PBS solution including 0.5% fluorescent beads with a diameter of 22 *μ*m, which is similar to that of the cells. The fluorescent beads were successfully captured in the trap array, as shown in Figure 7(a). In order to confirm the trap of a single cell, the cell flow via the microfluidic channel was observed using a microscope (Figure 7(b)), and the electrical impedance changes between the measurements with and without cell trapping were inspected. The impedance measurement of PBS at each site indicates that the variations (standard deviation) are less than 3.27%. The impedance difference between with and without a cell in the trap was approximately 10% at 5 kHz, indicating that a cell presence can be also inspected using electrical impedance.

There are other cell-capturing methods, such as chemical and electrical immobilization techniques. Chemical immobilization techniques have been utilized to trap ligands, proteins, and cells [19–21]. Although this method shows stable binding of the targets on a surface, the preliminary process of surface treatment using adhesion materials is necessary to immobilize the targets. In addition, a single cell positioning in the chemical immobilization appears to be difficult, compared to our physical cell immobilization method using a trap. A dielectrophoresis (DEP) force, which is one of the electrical immobilization techniques, has been used to capture a single cell in a nonuniform electric field [9]. This technique can realize label-free cell manipulation without the physical contact between the cell and electrodes [22]. However, the sensitivity for impedance measurement in our method seems to be relatively higher than the electrical immobilization technique using DEP force because the negative pressure in our device can lead to an electrical tight contact between a cell and the sensing electrodes. Although there are several merits of the trap for capturing cells compared to other cell immobilization techniques, the deformation of the cells due to the physical contact on the slanted side walls of the trap could affect the cell viability.

To the best of our knowledge, the present study is the first attempt to differentiate between normal and cancerous human urothelial cells in real time at an optimal frequency by applying the combination of the *μEIS-RT* with the *μEIS-OF*. A study using DEP chip which traps the cells in a microcavity by DEP force to measure the impedance of bladder cancer cell lines was reported in 2011 [9]. The idea of trapping bladder cancer cells prior to the impedance measurement was similar with the *μEIS-OF* part of our study. However, the trapping force of our *μEIS-OF* was experimentally estimated as five-times higher than the DEP chip. Moreover, our *μEIS-RT* part differentiates our work significantly from the referred study. It is expected that the DEP chip cannot properly capture every single cell in the specimen to measure their impedance, whereas our *μEIS-RT*, theoretically, can measure all of the cells promptly. As combining the *μEIS-RT* with the *μEIS-OF* in this study, our method is more compatible than the DEP chip in clinical application because it is possible to simply detect more cells in shorter time once the optimal frequency is obtained in advance. The data of the referred DEP chip study [9] and our study showed common results: the absolute value of amplitude and phase angle in the bladder cancer cells (T24 and TCCSUP) was lower than the normal cells (SV-HUC-1).

Instead of urothelial cells, a few studies have measured the electrical impedance of normal and cancerous urothelial tissues. A study reported in 2006 [23] used the Mk3.5 Sheffield System (2–384 kHz in 24 frequencies) to measure the impedance of bladder urothelial tissues. The results of this study show that the impedance significantly differs between the malignant and benign groups. A similar study was conducted in 2002 [24] using a custom-designed probe to measure the impedance at cystectomy specimens. The study shows that the* EIS* measurements could also significantly differentiate between benign and malignant tissues. These* EIS* studies using urothelial tissues were as effective as our study (using cells) in discriminating between the benign and malignant state. However, the tissue-level* EIS* studies require an invasive procedure with biopsy prior to the examination, whereas our *μEIS* device is expected to require only the voided urine specimen. The potential for noninvasiveness is one of the strongest advantages that our *μEIS* device has over the* EIS* which uses tissues.

In the present study, the cells measured by the *μEIS-RT* were fewer than we expected. Theoretically, as aforementioned, the *μEIS-RT* should not miss measuring the electrical impedance of cells passing the electrodes at a fixed frequency. However, despite the use of a cell suspension density of 1 × 10^6^/mL, the data could only be obtained from seventeen and twenty-seven SV-HUC-1 and TCCSUP cells, respectively. This low data acquisition was attributed to the high velocity of the cells passing through the *μEIS-RT* electrodes. The fastest sampling interval (12.5 ms) of the impedance analyzer used (HP 4294A; Agilent Technologies, Santa Clara, CA, USA) in the present study was much slower than the time that the cell spent passing the electrodes (below 2 ms). When the cells were infused into the device at a flow rate of 0.1 *μ*L/min, only the results shown above were obtained. A reduced flow rate of less than 0.1 *μ*L/min resulted in a lack of pressure for the cells to flow through the channel, and they could not approach the electrodes. The lowest flow rate allowing the cells to proceed through the channel is 0.1 *μ*L/min in the present study. This lowest flow rate was applied to maximize the detection rate of the cells as possible, whereas a higher flow rate led to the decrease in the cell detection rate compared to the flow rate of 0.1 *μ*L/min. For instance, the electrical signal of the cells was not detected at the maximum possible flow rate of 50 *μ*L/min. We believe that an impedance analyzer model with a faster sampling interval, such as the model “HF2LI” (Zurich Instruments, Zurich, Switzerland; fastest sampling interval: 2.2 *μ*s), could detect the signal of a large number of cells and increase the amount of acquired data despite the high cell flow velocity. Additionally, the maximal possible flow rate of the *μEIS* devices was 50 *μ*L/min (corresponding pressure: 84 kPa), and a higher flow rate led to the excessive internal pressure that bursts the device.

Urine specimens need to be tested with *μEIS* devices before application in the clinical field, and several requirements need to be met in order to conduct these tests. Because other substances in the urine specimen, such as red or white blood cells, may induce errors during urothelial cancer cell detection using the* EIS* devices, substances other than urothelial cells should be filtered out prior to the examination. A system that can collect urothelial cells from the whole urine volume from a patient is also necessary, because urothelial cancer cells may not be contained in a small sample of the specimen. Other improvements in *μEIS-RT* device could be made in the future to enhance detection sensitivity during flow cytometry. For example, a unit can be integrated into the device for preconcentrating bladder cells in the injected urine sample and removal of other impurities that hinder impedance response. Furthermore, studies of the matched electrical impedance values of urothelial cancer cells and their pathological grade would be useful to establish a treatment plan and predict the patient's prognosis.

## 5. Conclusions

The present study introduces the combination of two devices (*μEIS-OF* and *μEIS-RT*) to measure the electrical characteristics of normal and cancerous urothelial cell lines. The results imply that the *μEIS* can effectively differentiate between normal and cancerous human urothelial cells in real time at an optimal frequency on the basis of electrical impedance. Thus, the *μEIS* device has the potential to become a novel noninvasive technique to detect UCB using voided urine specimens and could be used to supplement cystoscopy and VUC after further development of the system along with more detailed studies.

## Figures and Tables

**Table 1:** Comparison ofelectrical impedance between the two cell lines in each *µEIS* device at 119 kHz.

Device type		TCCSUP	SV-HUC-1	*p*-value
*µEIS-OF*	Cell count	50	30	
	Mean amplitude (Ω)	13649.47 ± 93.22	14026.13 ± 105.58	<0.001
	Mean phase (°)	−80.57 ± 0.47	−82.39 ± 0.55	<0.001

*µEIS-RT*	Cell count	27	17	
	Mean amplitude (Ω)	14066.32 ± 91.54	14342.91 ± 108.59	<0.001
	Mean phase (°)	−85.85 ± 0.16	−86.25 ± 0.13	<0.001

*μEIS-OF*: micro-electrical impedance spectroscopy - optimal frequency; *μEIS-RT*: micro-electrical impedance spectroscopy - real-time; TCCSUP: urothelial cancer cell line; SV-HUC-1: normal urothelial cell line.
